# Prize Question

**Published:** 1817-04

**Authors:** 


*For the London Medical and Physical Journal.*

### *Prize Question.*

[We think it right to give the greatest publicity to the following, which we extract from the Philosophical Magazine.]

Z. of seventy-two years of age, of uniformly temperate and active habits, and of health in general good, though not robust, was afflicted twenty years ago with the jaundice, arising from gallstones which have not since troubled him; and three years ago, with a hernia humoralis, which suppurated, and left the patient considerably debilitated. Some time after this disease, his stomach became disordered, and has ever since been liable to loss of appetite, extreme flatulency, and frequent vomiting. This vomiting has usually occurred at night, about eight or ten hours after dinner. Sometimes, but rarely, food was ejected from the stomach. In general a bitter fluid, resembling bile, was thrown up. The violence of these symptoms sometimes intermitted for three or four weeks, and then returned again, as if from some noxious accumulation in the stomach.

Of course, the patient was weakened and somewhat ema-

 ciated ciated by the continuance of this disease; but his spirits, and the activity of his mind, have at no time been depressed. Every article of diet that is considered as flatulent had been at different times sedulously excluded. Animal and vegetable food have been tried alternately and in conjunction. Abstinence from wine, and from all fermented liquors, has also been tried; and these trials have been made with care by a person used to experiment, and whose appetite is entirely subservient to control; and, from repeated trials, he has found nothing which has appeared peculiarly obnoxious to his stomach, but fried fat, and the skinny or sinewy parts of meat, and the acetous acid.

At different times, wine of all sorts, and malt liquor, have been disagreeable to him. At other times, Port wine, mixed with twice the quantity of water, has been his beverage at dinner, with sometimes one, or at most two, small glasses of Port wine after dinner. But malt liquor, viz. small beer of a good quality, has seemed to agree with him better than any other liquid. Water, which in his neighbourhood is excellent, has not been found salutary—for whether water, beer, or wine, or meat, or vegetables, or simple puddings, or roasted apples, made part of his diet, the same inordinate flatulency and sometimes subsequent vomiting have recurred. It is to be observed, the acidity which usually attends common indigestion has not been a symptom in this disease.

The bowels have in general been kept in a proper state by a small quantity of aloes taken in the form of Darwin's dinner pills, which has never produced any constriction nor any excess.

Various remedies have been proposed by the medical friends of the patient, who is happily known to many men of medical eminence. Opium, bitters, alkalies have been tried; but every kind of drug seems to have been ineffectual. Indeed, the patient, though he has most sincere regard for individuals of the medical profession, and a high opinion of their skill, is not at all inclined to tamper with his constitution.

There is another symptom attendant upon this disease, which must be mentioned. Ever since his recovery from the hernia humoralis, three years ago, his urine has never returned to its natural colour, but has constantly been of a wheyish appearance, yet without flocculi or gluten. It has been voided in usual quantities in the day-time, but with unusual frequency in the night. At times, the urinary secretion has been affected by a strange propulsion of wind from the urethra, attended with a considerable hissing or explosive noise, and ejecting a white fluid that has the exact appearance appearance of cuckoo-spittle; but which, on being touched, appears to be nothing but common urine mixed with air, without any gelatinous consistence. This symptom is always attended with a slight distension of the rectum, so that the patient cannot determine the source from whence this gas is evolved; but this affection is not attended with any pain or inconvenience.

The pulse has usually been regular, beating from 72 to 86 in a minute; at times, however, it has intermitted, and totally stopt for some seconds; but this irregularity has of late been less frequent.

At the beginning of dinner, a spoonful or two of soup, or the first morsel he eats, particularly if salt or spiced, produces a profuse perspiration on the head, though at other times neither heat nor exercise produces perspiration.

No prescription or advice will be attended to, the materials or rationale of which are concealed.

***** I have to state, that a premium is offered by a gentleman of honour, and that I will take care to forward to his address any communications which medical gentlemen may wish to send to him. Let them be sent sealed, and without expense, in an envelope, stating that the enclosure relates to the premium.—A.T. [Alexander Tilloch, the Phil. Mag. is published by Cadell and Davis.]**

